# The association between the upper digestive tract microbiota by HOMIM and oral health in a population-based study in Linxian, China

**DOI:** 10.1186/1471-2458-14-1110

**Published:** 2014-10-27

**Authors:** Guoqin Yu, Bruce A Dye, Mitchell H Gail, Jianxin Shi, Vanja Klepac-Ceraj, Bruce J Paster, Guo-Qing Wang, Wen-Qiang Wei, Jin-Hu Fan, You-Lin Qiao, Sanford M Dawsey, Neal D Freedman, Christian C Abnet

**Affiliations:** Division of Cancer Epidemiology and Genetics, National Cancer Institute, National Institutes of Health, Rockville, MD USA; Centers for Disease Control and Prevention/National Center for Health Statistics, Hyattsville, MD USA; Forsyth Institute, Cambridge, MA USA; Wellesley College, Wellesley, MA USA; Harvard School of Dental Medicine, Boston, MA USA; Cancer Institute, Chinese Academy of Medical Sciences, Beijing, PR China; Genetic Epidemiology Branch, Division of Cancer Epidemiology and Genetics, National Cancer Institute, 9609 Medical Center Dr Rm 6E518 MSC 9704, Bethesda, MD 20892-9769 USA

**Keywords:** Microbiota, Oral health, Dental caries, Periodontitis, Bleeding on probe, Attachment loss

## Abstract

**Background:**

Bacteria affect oral health, but few studies have systematically examined the role of bacterial communities in oral diseases. We examined this relationship in a large population-based Chinese cancer screening cohort.

**Methods:**

Human Oral Microbe Identification Microarrays were used to test for the presence of 272 human oral bacterial species (97 genera) in upper digestive tract (UDT) samples collected from 659 participants. Oral health was assessed using US NHANES (National Health and Nutrition Examination Survey) protocols. We assessed both dental health (total teeth missing; tooth decay; and the decayed, missing, and filled teeth (DMFT) score) and periodontal health (bleeding on probing (BoP) extent score, loss of attachment extent score, and a periodontitis summary estimate).

**Results:**

Microbial richness, estimated by number of genera per sample, was positively correlated with BoP score (P = 0.015), but negatively correlated with tooth decay and DMFT score (P = 0.008 and 0.022 respectively). Regarding β-diversity, as estimated by the UniFrac distance matrix for pairwise differences among samples, at least one of the first three principal components of the UniFrac distance matrix was correlated with the number of missing teeth, tooth decay, DMFT, BoP, or periodontitis. Of the examined genera, *Parvimonas* was positively associated with BoP and periodontitis. *Veillonellacease* [G-1] was associated with a high DMFT score, and *Filifactor* and *Peptostreptococcus* were associated with a low DMFT score.

**Conclusions:**

Our results suggest distinct relationships between UDT microbiota and dental and periodontal health. Poor dental health was associated with a less microbial diversity, whereas poor periodontal health was associated with more diversity and the presence of potentially pathogenic species.

**Electronic supplementary material:**

The online version of this article (doi:10.1186/1471-2458-14-1110) contains supplementary material, which is available to authorized users.

## Background

The human mouth harbors one of the most diverse microbial communities (microbiotas) in the human body with over 700 bacterial species identified [1, 2]. The oral microbiota is essential for the development of the mucosal immune system, the maintenance of a normal physical environment, and for the digestion of food [3]. Loss or imbalance of normal microbiota may lead to an overgrowth of opportunistic bacterial pathogens, leading to disease [4].

The oral microbiota is intimately related to oral health [5]. Dental caries occur when organic acids from bacterial fermentation of carbohydrates demineralize the teeth [6]. Periodontitis, the inflammatory response in the gingival tissue and surrounding connecting tissue, can result in the loss of collagen attachment between the tooth and the bone, leading to bone destruction and tooth loss [7]. The majority of oral microbiology studies have focused on individual candidate pathogens that could be cultured [8]. However, oral diseases may be related to the diversity and function of the entire oral microbial community, not just single species [5]. With the introduction of culture independent technology, researchers can now investigate the role of the entire oral microbial community [8–12]. However, the majority of such studies [8–12] have been small and have often focused on single oral diseases and people with good access to dental care.

Culture-independent approaches, like 16S rRNA gene-based methods can provide a comprehensive view of the human microbial communities, including currently un-culturable microorganisms. In this study, we used a 16S rRNA gene-based microarray method (the Human Oral Microbe Identification Microarray) to examine the relationship between the upper digestive tract (UDT) microbiota and oral health (including both dental and periodontal health) in 659 healthy Chinese adults from a cancer screening study in Linxian, China, a rural cohort with poor access to dental care and a high rate of esophageal cancer.

## Methods

### The Study Cohort

Study participants were enrolled from three villages in Linzhou (formally Linxian), Henan Province, People’s Republic of China. The description of the parent study (the Cytology Sampling Study 2, CSS2) was published previously [13]. Participants were apparently healthy volunteers, aged 40–65 years [13]. We successfully obtained UDT microbiota data in upper digestive tract samples from 659 out of the original 720 study participants. The missing of UDT microbiota data were at random due to failure in DNA extraction or microarray. This study was conducted under the auspices of the Institutional Review Boards of the Cancer Institute, Chinese Academy of Medical Sciences and the US National Cancer Institute, and all subjects provided written informed consent.

### Sample collection, DNA extraction

Upper digestive tract (UDT) cell samples for DNA extraction were collected by one of two esophageal balloon cytology retrieval devices. One consisted of an inflatable rubber balloon covered with cotton mesh attached to a 0.2 cm diameter single lumen rubber tube (manufactured in China, and called the Chinese balloon type (CHB)). The second device was the Cytomesh Esophageal Cytology Device (WCH) (Wilson-Cook Medical, Inc, Winston-Salem, NC, USA). Allocation of the sampling devices was random. Each volunteer was given 2 ml of 2% lidocaine slurry by mouth, after which the balloon was inserted into the stomach, inflated, drawn through the esophagus, deflated at the upper esophageal sphincter, and withdrawan from the mouth. The head of the balloon was cut off and deposited in 40 ml of sterile saline in a 50 ml centrifuge tube and transferred to the lab on ice for processing. This centrifuge tube was then vortexed for 30 seconds, the balloon head was removed, and the remaining fluid was centrifuged at 1500 RPM for 5 minutes. After discarding the supernatant, the pellet was re-suspended in 1 ml saline, frozen in liquid nitrogen, and stored at -80 ° C until DNA extraction.

DNA was extracted by the Gentra Puregene Cell Kit (Qiagen, Valencia, CA). The quality and quantity of the DNA was checked by the 260:280 ratio, nanodrop, and picogreen. The presence of human and bacterial DNA was verified by TaqMan with species-specific primers.

### DNA preparation for array hybridization and HOMIM array

The Human Oral Microbe Identification Microarray (HOMIM) analysis was conducted in the Paster laboratory as previously described [14]. The array uses 16S rRNA-based oligonucleotide probes printed on glass slides. The extracted DNA was used as the template in PCR reactions with universal forward and reverse primers and labeled in a second nested PCR. Bacterial 16S rRNA gene amplicons from the UDT samples were hybridized onto 16S rRNA gene microarrays [14]. After hybridization, the washed slides were scanned using GenePix Pro software. A normalized median intensity score was generated by subtracting the median feature intensity from the background intensity for each individual feature. The relative intensity of each probed species/strain was then estimated using feature-specific criteria.

HOMIM array was developed to profile human oral microbiota. Thus, we are studying oral bacterial species in the UDT samples although the UDT samples include cells from the stomach, esophagus and mouth.

### Estimation of oral health parameters

The National Health and Nutrition Examination Survey (NHANES) oral health examination protocols were used to obtained information on oral health [15]. Dental health was assessed by the number of missing teeth (total permanent teeth missing, including third molars), the amount of untreated tooth decay (the total surfaces of all teeth with coronal decay), and the DMFT score (decayed, missing, and filled teeth, excluding third molars). Periodontal health information was assessed by bleeding on probing (BoP, the percent of probed sites with bleeding), attachment loss (AL, the percent of probed sites with loss of attachment > = 3 mm), and a periodontitis summary estimate (presence/absence of periodontal disease, present if one or more probed periodontal site had an attachment loss of 3 mm or greater and a pocket depth of 4 mm or greater). Assessments of untreated tooth decay, DMFT, bleeding on probing and attachment loss were restricted to participants with at least one tooth (dentate). Four examiners independently conducted the clinical visits and the details of examiner calibration were described previously [15].

### Statistical analysis

As some of the probes on the arrays hybridized to more than one species, microbial richness was estimated as the number of genera in each sample. β-diversity, which estimates pairwise difference in microbial diversity between samples, was measured by constructing an unweighted UniFrac distance matrix via FastUniFrac (http://unifrac.colorado.edu) [16]. The phylogeny used for UniFrac estimation was constructed from Human Oral Microbiome Database [17]. The unweighted UniFrac matrix was analyzed by principle coordinate (PC) analysis. The first three PCs, which explain respectively 22%, 14%, 9% of the variance, were used for further analyses.

Associations between number of genera per sample, the first three PCs for β-diversity and oral health variables were evaluated by adjusted linear regression models and Spearman tests in R [18]. Both linear regression models and Spearman tests were adjusted for age (years), sex (female or male), history of smoking (yes or no), history of antibiotic use in the last three months (yes or no), and sample device (CHB or WCB). Adjusted Spearman tests used the residuals of each health variable and residuals of each microbial variable after linearly fitting them against the above adjustment variables. We used nonparametric Spearman tests due to the non-normal distribution of each oral health variable. A statistical significance level of 0.05 was applied. However we did not show Spearman tests results in the tables because they produced similar results. We conducted several sensitivity analyses including stratifying the analysis by sampling device and by removing patients that reported taking antibiotics in the last 3 months prior to enrollment.

To examine the relationship between oral health and the presence/absence of a specific genus, we used logistic regression models. Bonferroni correction was used to account for multiple testing.

## Results

### Characteristics of the study cohort

Table 1 describes the study cohort. Among the study participants, the average age was 55 years, 42% were male, 25.5% were never-smokers, and 11.5% had used antibiotics in the last three months. With regard to oral health, although most had no tooth decay (median = 0), 17.5% of participants had lost all of their teeth, and 43% of participants had periodontitis.

**Table 1:** **The distribution of demographic and study characteristics and oral health parameters of the Cytology Sampling Study 2 subjects evaluated in this study**

	N (%)	Mean (SD)	Median (range)
Age		54.94(4.91)	54.00(34.00-67.00)
Gender			
Male	279(42.3%)		
Female	380(57.7%)		
Smoking			
Yes	491(74.5%)		
No	168(25.5%)		
Antibiotic usage in the last three months			
Yes	76(11.5%)		
No	583(88.5%)		
Balloon sampling device			
Chinese balloon	320(48.6%)		
Wilson-Cook balloon	339(51.4%)		
**Dental health parameters**			
Teeth missing^a^		12.64(11.27)	8.00(0.00-32.00)
Teeth decay^b^		1.59(2.88)	0.00(0.00-22.00)
DMFT^c^		11.89(10.00)	9.00(0.00-28.00)
Presence of teeth			
At least one teeth present	544(82.5%)		
No teeth present	115(17.5%)		
**Periodontal health**			
BoP^d^		0.57(0.27)	0.57(0.00-1.00)
AL^e^		0.44(0.31)	0.40(0.00-1.00)
Periodontitis^f^			
Present	227(43.0%)		
Absent	301(57.0%)		

^a^Number of permanent teeth missing.

^b^Total surfaces with coronal decay.

^c^DMFT (decayed, missing, and filled teeth) score based on teeth excluding third molars.

^d^Bleeding of probe extent score, percent of probed sites with bleeding.

^e^Loss of attachment extent score, percent of probed sites with loss of attachment > =3 mm.

^f^Periodontitis defined as present if one or more probed periodontal site had an attachment loss of 3 mm or greater and a pocket depth of 4 mm or greater.

### Associations between oral health variables and microbial richness

In both adjusted linear regression models and Spearman correlation tests, microbial richness, measured as number of genera per sample, was associated inversely with several measures of poor dental health (teeth missing, tooth decay, and DMFT score), but was associated positively with poor periodontal health (BoP, AL, and periodontitis). Many of these associations were statistically significant (Table 2). For example, increased tooth decay and DMFT score were significantly associated with fewer genera per sample (P_linear_ = 0.01, 0.02, respectively), and increased BoP was significantly associated with more genera per sample (P_linear_ = 0.02, P_Spearman_ = 0.02).

**Table 2:** **Association between oral health parameters and microbial richness measured as number of genera per sample**

	Coefficient	p value
**Dental health**		
Teeth missing^a^	-0.093 ± 0.068	0.175
Tooth decay^b^	**-0.066 ± 0.021**	**0.008**
DMFT^c^	**-0.138 ± 0.060**	**0.022**
Presence of teeth^d^	0.025 ± 0.018^h^	0.175^h^
**Periodontal health**		
BoP^e^	**0.005 ± 0.002**	**0.015**
AL^f^	0.003 ± 0.002	0.110
Periodontitis^g^	0.020 ± 0.015^h^	0.162^h^

^a^Number of permanent teeth missing.

^b^total surfaces with coronal decay.

^c^DMFT score (decayed, missing, and filled teeth) based on teeth excluding third molars.

^d^Compare the number of genera in subjects with and without complete loss of teeth.

^e^Bleeding of probe extent score, percent of probed sites with bleeding.

^f^Loss of attachment extent score, percent of probed sites with loss of attachment > =3 mm.

^g^Comparing those with and without periodontitis by the periodontitis summary estimate (logistic model).

^h^Coefficient and p value based on adjusted logistic regression model.

All linear and logistic models adjusted for age, gender, smoking status, antibiotic use in the last 3 months and sampling device type.

Bolded if P value is less than 0.05.

We found no significant difference in microbial richness between toothless subjects and those with one or more teeth remaining (Table 2).

### Significant association between Oral health variables and microbial β-diversity

We assessed associations between β-diversity and oral health using the first three principal components (PCs) of the unweighted UniFrac distance matrix (Table 3). Of the six tested oral health measures, five of them were associated with at least one of these three PCs. Tooth decay was associated with PC1 (P_linear_ = 0.01, P_spearman_ = 0.05). Teeth missing and BoP were associated with PC2 (teeth missing, P_linear_ = 0.01, P_Spearman_ = 0.01; BoP, P_linear_ = 0.01, P_spearman_ = 0.01). DMFT score and periodontitis were significantly associated with PC2 and PC3. PC 2 and PC3 were also associated with whether or not participants had any teeth.

**Table 3:** **Association between oral health parameters and microbial β diversity**

	PC1		PC2		PC3	
	Coefficient	p	Coefficient	p	Coefficient	p
**Dental health**						
Teeth missing^a^	0.96 ± 3.14	0.76	**-11.84 ± 3.88**	**0.01**	8.44 ± 4.89	0.09
Tooth decay^b^	**2.90 ± 0.99**	**0.01**	-1.98 ± 1.25	0.12	1.23 ± 1.56	0.43
DMFT^c^	3.70 ± 2.77	0.18	**-11.34 ± 3.42**	**0.01**	**8.37 ± 4.32**	**0.05**
Presence of teeth^d^	-0.18 ± 0.81^h^	0.83^h^	**2.08 ± 1.00** ^**h**^	**0.04** ^**h**^	**-3.80 ± 1.42** ^**h**^	**0.01** ^**h**^
**Periodontal health**						
BoP^e^	-0.12 ± 0.09	0.17	**0.33 ± 0.11**	**0.01**	-0.24 ± 0.14	0.07
AL^f^	-0.10 ± 0.10	0.34	0.03 ± 0.13	0.83	-0.14 ± 0.16	0.38
Periodontitis^g^	-0.33 ± 0.66^h^	0.63^h^	**2.28** ± **0.86** ^h^	**0.01** ^h^	**-2.08** ± **1.05** ^h^	**0.05** ^h^

^a^Number of permanent teeth missing, including third molars.

^b^Total surfaces with coronal decay.

^c^DMFT (decayed, missing, and filled teeth) score, based on teeth excluding third molars.

^d^Comparing those subjects with and without any remaining teeth (logistic model).

^e^Bleeding of probe extent score, percent of probed sites with bleeding.

^f^Loss of attachment extent score, percent of probed sites with loss of attachment > =3 mm.

^g^Comparing those with and without periodontitis by the periodontitis summary estimate (logistic model).

^h^Coefficient and p value based on adjusted logistic regression model.

All linear and logistic models adjusted for age, gender, smoking status, antibiotic use in the last 3 months and sampling device type.

Bolded if the P value is less than 0.05.

In addition, we used the Unweighted Pair Group Method with Arithmetic Mean (UPGMA) to create clusters based on the UniFrac distance matrix (Additional file 1). We focused on clusters B and C, as cluster A included only 11 participants. However, such clusters were not associated with any of our examined oral health measures (Additional file 2).

### Association between oral health and presence/absence of specific genera

We also examined associations between the oral health variables and the presence/absence of specific genera (Additional file 3). Table 4 showed the associations that remained significant after Bonferroni correction. Subjects with *Parvinomas* had a significantly higher risk for periodontitis (p = 0.000219). Subjects with *Parvimonas* or *Porphyromonas* had a significantly higher extent of BoP (P = 0.000006 and 0.0002, respectively). Subjects with *Filfactor* or *Peptostreptococcus* had a significantly lower DMFT score (P = 0.0000006 and 0.000005, respectively) while subjects with *Veillonellaceae [G-1]* had a significantly higher DMFT score (P = 0.00009).

**Table 4:** **The list of genera whose presence was significantly associated with oral health variables after Bonferroni correction**

		Statistics of oral health variables for the subjects with or without the genus			Logistic model	
Oral health variables	Genus	Number (proportion) of samples with the genus	Variable value in subjects with the genus (median, IQR)	Variable value in subjects without the genus (median, IQR)	Coefficient	P value
Periodontitis	*Parvimonas*	411 (0.78)	0.47^c^	0.28^c^	0.852	2.2E-04
BoP^a^	*Parvimonas*	411 (0.78)	0.60(0.40-0.79)	0.44(0.27-0.67)	1.041	5.9E-06
	*Porphyromonas*	268 (0.51)	0.61(0.41-0.82)	0.55(0.33-0.70)	1.065	2.3E-04
DMFT^b^	*Filifactor*	238 (0.36)	4(2-9)	9(3-21)	-0.048	6.0E-07
	*Peptostreptococcus*	105 (0.16)	5(2-10)	10(4-24)	-0.065	4.7E-06
	*Veillonellaceae [G-1]*	216 (0.33)	11(6-28)	7(2-17)	0.036	9.0E-05

IQR, interquartile range.

^a^Bleeding on probing, percent of probed sites with bleeding.

^b^DMFT (decayed, missing, and filled teeth) score based on teeth excluding third molars.

^c^Proportion of samples with periodontitis.

## Discussion

In this study, we examined several aspects of UDT microbiota, including microbial richness, β-diversity, and specific bacterial genera. Each aspect was associated with one or more measures of poor dental or periodontal health, although these associations differed. Poor dental health was associated inversely with microbial richness, and certain bacterial species were notably absent among those with high DMFT scores. In contrast, poor periodontal health was associated positively with microbial richness and the presence of species that have previously been implicated in this disease.

An integral part of the tooth decay process is demineralization of the tooth structure, which can lead to the development of cavities. Untreated tooth decay can result in substantial loss of tooth structure, infection, and pain. We found significant inverse associations between microbial richness, tooth decay, and DMFT score. Our findings are concordant with the ecological plaque hypothesis [19], which suggests that caries stem from a shift in the balance of UDT microbiota in response to environmental pressures, such as frequent carbohydrate intake or acidification of the oral environment, and that these changes lead to an overall reduction of microbial richness.

Alternatively, two other hypotheses have been described: the specific plaque hypothesis and the nonspecific plaque hypothesis [20, 21]. The specific plaque hypothesis proposes that only a few specific species, such as *Streptococcus mutans* and *Streptococcus sobrinus*, are actively involved in the disease [20]. On the other hand, the nonspecific plaque hypothesis suggests that caries arise from the combined actions of the many different bacterial species residing in the plaque [21]. Carries, according to this hypothesis, may be caused by increasing presence of acid-producing oral micro-organisms and/or a decrease in alkali-producing bacteria. Consistent with the nonspecific hypothesis, recent studies have suggested the involvement of many different genera, including *Lactobacillus*, *Actinomyces, Bifidobacterium, Veillonella, Corynebacterium* or *Leptotrichia* [22, 23], in this process*.* Some of these bacteria, particularly *Veillonella*, have been shown to be predominant at all stages of caries progression [22] and under high-glucose conditions, and have been implicated in acid production [24]. In our study, we found that subjects with a high DMFT score were more likely to have *Veillonellaceae [G-1*], but less likely to have *Filifactor* and *Peptostreptococcus*. As *Veillonellaceae [G-1]* grows well in an acid environment, and *Peptostreptococcus* does better in a basic environment [25], our results are consistent with the non-specific plaque hypothesis.

BoP is an indicator of periodontal tissue inflammatory response to bacterial pathogens and may indicate active periodontitis, whereas attachment loss reflects the loss of supporting tissue as part of the periodontal disease process. We found a significant positive association between BoP and microbial richness and a consistent, though non-significant, association between attachment loss and periodontitis. Our results are consistent with previous findings [9, 26]. The association of increased microbial richness and increased BoP suggests that pathogenic periodontal species may colonize the mouth during periods of periodontal inflammation. Changes in the microbial community during periodic inflammatory events within the periodontium may be a progressive process, with the introduction of taxa associated with inflammation without the replacement of original resident species.

Previous studies suggest that several key species are associated with periodontitis [27–31]. These species include *Porphyromonas gingivalis, Treponema denticola, Prevotella intermedia, Aggregatibacter actinomycetemcomitans,* and *Fusobacterium nucleatum.* Culture-independent approaches, such as quantitative ribosomal 16S cloning and sequencing techniques have implicated other species including *Parvimonas micra*, *Filifactor alocis,* uncultivated clones from the *Deferribacteres* and *Bacteroidetes* phyla, *Megasphaera* clone BB166, and clone I025 from the TM7 phylum [32, 33]. Our results provide further support for the role of *Porphyromonas* and *Parvimonas* in periodontitis as previous studies suggested [34–38]. However, it remains unclear whether these bacterial species are responsible for initiation of the disease or whether periodontitis creates conditions that select for these bacteria. Studies with prospective designs and experiments on animal models are required.

Another important finding was the observed significant association between β-diversity and most of our examined health measures, highlighting the important association between the UDT microbiota and oral health. Previous studies of the gut microbiota have shown that diet, race, and geography contribute to the difference among gut microbial communities [39–41]. Our findings suggest that oral health makes a similarly important contribution to the UDT microbiota.

Our study has several strengths and limitations. The strengths include the relatively large sample size and the availability of detailed oral health and demographic information on participants. It is one of only a few studies that have focused on a population with poor access to dental care and have had potential confounders measured. The limitations include the cross-sectional design of the parent study, and the use of the HOMIM array with samples from a device that collected cellular and luminal material from the stomach, esophagus, and oral cavity. The HOMIM arrays are specifically designed to measure oral bacteria, and therefore did not allow us to study bacterial species not on the array. Therefore, the results may reflect only the oral bacteria and these bacteria when present in the esophagus and stomach.

## Conclusions

Our study suggests that tooth decay and DMFT score were associated with a microbial community shift towards decreased microbial richness, which is likely due to an increase of aciduric and decrease of alkali-producing bacteria species. Participants with periodontitis had a distinct microbial community, characterized by increased microbial richness and the presence of additional, potentially pathogenic, bacterial genera such as *Porphyromonas* and *Parvimonas*. However, due to the cross-sectional design, we could not determine whether these changes in the UDT microbiota were the cause or the result of oral disease. Experimental studies and prospective cohorts with repeated assessment are needed to define the temporal relationship between microbial communities and oral health.

## Electronic supplementary material

Additional file 1: **Subjects grouped into clusters (designated as A, B, C) by the UniFrac distance matrix according to the UPGMA method.** (PDF 32 KB)

Additional file 2: **Comparisons of oral health variables between subjects in clusters B and C from the UPGMA analysis of the UniFrac distance matrix (clusters shown in Additional file** 1 **).** (DOC 33 KB)

Additional file 3: **Association between each oral health variable and presence of each genus in HOMIM array.** (XLS 54 KB)

## Abbreviations

UDT: Upper digestive tract
NHANES: National Health and Nutrition Examination Survey
HOMIM: Human Oral Microbe Identification Microarray
DMFT: Decayed, missing, and filled teeth score based on teeth excluding third molars
BoP: Bleeding of probe extent score, percent of probed sites with bleeding
AL: Loss of attachment extent score, percent of probed sites with loss of attachment > = 3 mm.
