# Supplementary figures and images for: The association between the upper digestive tract microbiota by HOMIM and oral health in a population-based study in Linxian, China
Source: BMC Public Health. 2014 Oct 27;14:1110. doi: 10.1186/1471-2458-14-1110 (PMC4223728; doi:10.1186/1471-2458-14-1110)

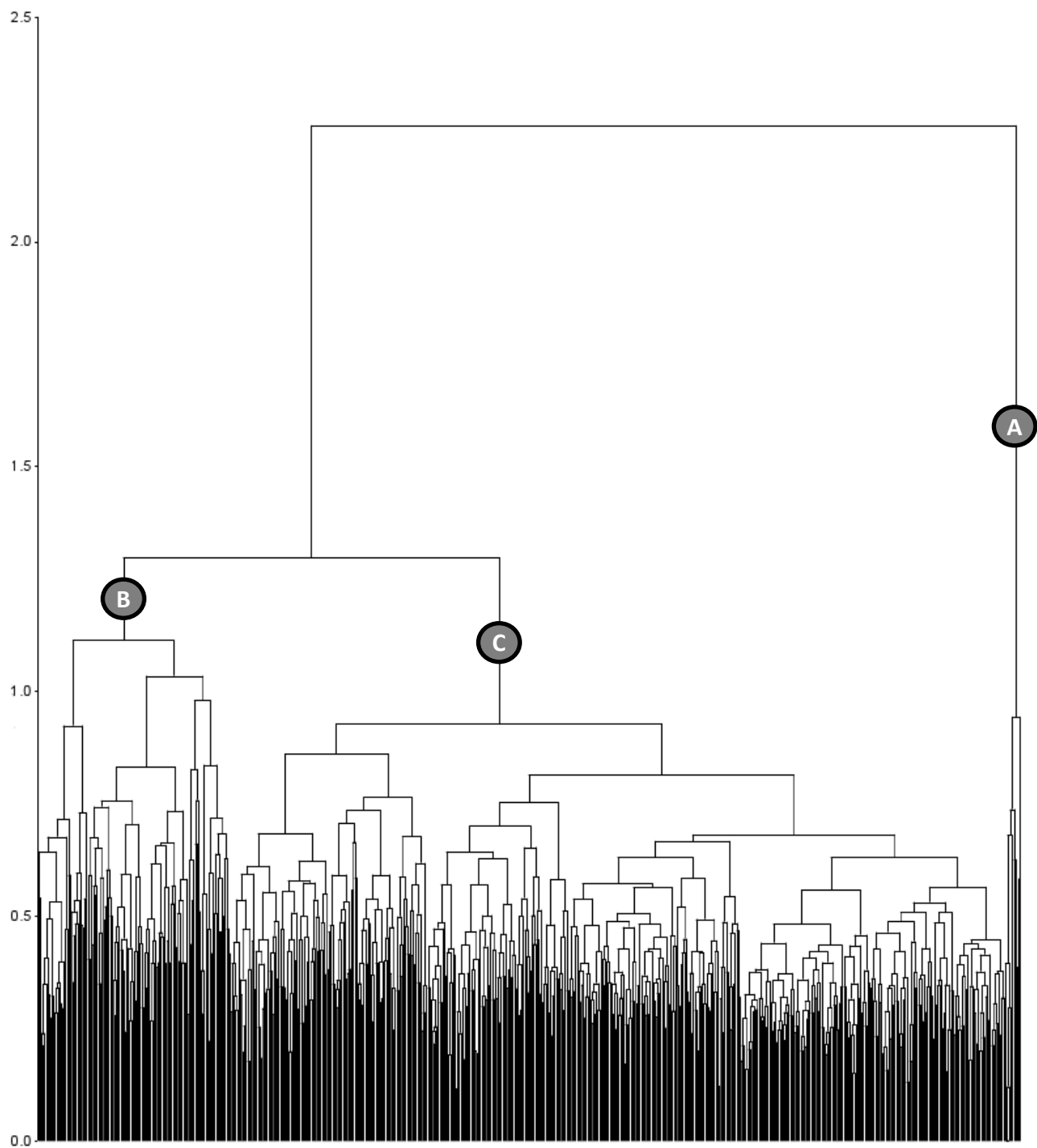

Supplement: Supplementary file 1 — Additional file 1: Subjects grouped into clusters (designated as A, B, C) by the UniFrac distance matrix according to the UPGMA method. (PDF 32 KB) [file 12889_2014_7206_MOESM1_ESM.pdf]
