# Supplementary material for: The association between the upper digestive tract microbiota by HOMIM and oral health in a population-based study in Linxian, China
Source: BMC Public Health. 2014 Oct 27;14:1110. doi: 10.1186/1471-2458-14-1110 (PMC4223728; doi:10.1186/1471-2458-14-1110)
Supplement: Supplementary file 2 — Additional file 2: Comparisons of oral health variables between subjects in clusters B and C from the UPGMA analysis of the UniFrac distance matrix (clusters shown in Additional file 1 ). (DOC 33 KB) [file 12889_2014_7206_MOESM2_ESM.doc]

**Additional file 2,** **Comparisons of oral health variables between subjects in clusters B and C from the UPGMA analysis of the UniFrac distance matrix (clusters shown in Supplementary Figure 1)**

|  | **Median (IQR)** | |  |  |
| --- | --- | --- | --- | --- |
|  | **cluster B (n=519)** | **cluster C (n=129)** |  | **P value^g^** |
| Teeth missing^a^ | 8(4-20) | 8(4-22) |  | 0.82 |
| Tooth decay^b^ | 1(0-3) | 1(0-2) |  | 0.87 |
| DMFT^c^ | 9(3-19) | 10(3-23) |  | 0.89 |
| BoP^d^ | 0.571(0.367-0.769) | 0.583(0.362-0.760) |  | 0.89 |
| AL^e^ | 0.417(0.174-0.691) | 0.361(0.143-0.686) |  | 0.74 |
| percent of subjects with, % |  |  |  |  |
| Periodontitis^f^ | 54 | 57 |  | 0.63 |
| At least one teeth | 83 | 82 |  | 0.69 |

^a^ Number of permanent teeth missing;

^b^ total surfaces with coronal decay;

^c^ DMFT (decayed, missing, and filled teeth) score based on teeth excluding third molars;

^d^ Bleeding of probe extent score, percent of probed sites with bleeding;

^e^ Loss of attachment extent score, percent of probed sites with loss of attachment >= 3 mm;

^f^ Periodontitis defined as present if one or more probed periodontal site had an attachment loss of 3 mm or greater and a pocket depth of 4 mm or greater;

^g^ P value based on the logistic regression model adjusted for the following potential confounders including age, gender, smoking status, antibiotic usage in the last 3 months and sampling device
